# Clinical and injury-related factors associated with specific postoperative complications after surgical treatment of distal radius fractures: a retrospective observational study

**DOI:** 10.1186/s12893-026-03887-z

**Published:** 2026-06-12

**Authors:** Mingyang Yuan, Xiaotian Ma, Zheng Xu

**Affiliations:** 1 Trauma Surgery/Emergency Surgery, Affiliated Hospital of Jiangnan University, No. 1000, He Feng Road, Binhu District, Wuxi, Jiangsu Province 214122 China; 2 Trauma and Orthopedics Department, Zhangjiagang First People’s Hospital, No. 68, Jiyang West Road, Zhangjiagang City, Suzhou, Jiangsu Province 215600 China

**Keywords:** Distal radius fracture, Postoperative complications, Risk factors, Retrospective study, Rehabilitation compliance

## Abstract

**Background:**

With an ageing population and increased physical activity, distal radius fractures (DRF) are relatively common. Surgical treatment is becoming increasingly prevalent, yet postoperative complications including complex regional pain syndrome (CRPS), tendon irritation/rupture, carpal tunnel syndrome, internal fixation‑related discomfort, and non‑union/malunion significantly impact functional recovery and quality of life. Identifying associated risk factors is crucial for optimising treatment strategies.

**Objective:**

To investigate the clinical and injury-related risk factors for specific postoperative complications in patients undergoing surgical treatment for distal radius fractures.

**Methods:**

A retrospective observational study was conducted. Medical records of patients undergoing surgical treatment for DRF at our hospital between January 2022 and June 2025 were collected. Patients were categorised into a complication group and a non-complication group based on the occurrence of specific postoperative complications. Propensity score matching (PSM) was used to balance baseline differences, resulting in 60 cases per group. Primary outcomes comprised the incidence of specific postoperative complications and independent risk factors. Secondary outcomes included wrist range of motion, grip strength, and DASH (Disabilities of the Arm, Shoulder, and Hand) scores at 6 months postoperatively; injury-related factors [AO (Arbeitsgemeinschaft für Osteosynthesefragen) fracture classification, open fracture status, time from injury to surgery]; surgical factors (surgical approach, operative time, use of internal fixation); postoperative complications (diabetes mellitus, osteoporosis); postoperative management (immobilisation duration); and rehabilitation protocols. Univariate and multivariate logistic regression analyses were employed to identify risk factors.

**Results:**

After PSM, 60 cases and 60 controls were analysed. Among the 60 cases, the most common complication types were tendon irritation/rupture (31.7%) and internal fixation-related discomfort (23.3%), followed by carpal tunnel syndrome (20.0%) and CRPS (13.3%). Non-union/malunion occurred in 11.7% of cases. These proportions reflect the distribution of complication subtypes within the case group and do not represent population incidence rates. Multivariate analysis revealed that an operative time exceeding 90 min (OR = 4.006, 95% CI: 1.806–3.888) constituted an independent risk factor for postoperative complications (*p* < 0.05). Analysis of secondary outcomes revealed that the complication group exhibited significantly poorer wrist flexion/extension range of motion, percentage of grip strength recovery, and DASH scores at 6 months postoperatively compared to the non-complication group (*p* < 0.05). The incidence rates of all three major complications exhibited a significant increasing trend with higher fracture classification grades (*p* < 0.05). No statistically significant differences were observed between the complication and non-complication groups regarding gender, timing of surgery, approach selection, or type of internal fixation (*p* > 0.05).

**Conclusion:**

The occurrence of specific complications following distal radius fracture surgery is closely associated with multiple factors, including surgical technique, injury severity, and postoperative management. The key finding is that operative time > 90 min was strongly associated with complications (OR 4.01), but this association likely reflects underlying case complexity rather than a direct causal effect. Fracture severity (AO-C) and early rehabilitation showed only associative patterns that were either confounded or bidirectional. Thus, the study’s primary contribution is to highlight the need for caution in interpreting operative time as a modifiable risk factor without adjusting for complexity, and to identify key unmeasured confounders for future research.

## Introduction

Distal Radius Fracture (DRF) is one of the most common orthopedic injuries worldwide, and its epidemiological data reveal the significant burden of this disease [1, 2]. Among the elderly population, especially post-menopausal women, it is closely related to osteoporosis and is the most common type of fracture after low-energy falls [3, 4]. In the younger population, high-energy trauma such as sports injuries or traffic accidents become the main causes of injury [5, 6]. With the acceleration of the aging process of society and the general increase in public expectations for wrist joint function recovery, the proportion of surgical treatment for DRF is showing a continuous upward trend [7, 8]. Open reduction and internal fixation, especially the palmar locking plate technique, as it can provide stable anatomical reduction and allow early functional exercise, has become the mainstream choice for treating unstable or intra-articular fractures [9, 10]. This transformation in the treatment model has significantly improved the prognosis of patients, shortened the recovery period, and also pushed the identification and management of postoperative complications to the forefront of clinical attention.

Despite the continuous improvement of surgical techniques, the occurrence of postoperative complications remains an inevitable reality that cannot be completely avoided, and its impact extends far beyond merely prolonging the recovery time [11]. Specific postoperative complications—including complex regional pain syndrome (CRPS), tendon irritation/rupture, carpal tunnel syndrome, non-union/malunion, and internal fixation-related discomfort—may lead to persistent pain and impaired wrist function. In severe cases, these complications even require secondary surgical intervention, ultimately impairing long-term functional outcomes and quality of life [12, 13]. Although existing literature has explored the individualized risk factors for various complications, such as advanced age, degree of fracture comminution, and duration of surgery, the conclusions often show inconsistency [14]. Many studies have analyzed various complications in a general manner, ignoring the fact that different complications may have distinct pathological mechanisms and risk spectra; some studies, due to limited sample size or insufficient control of confounding variables, have restricted the reliability of their conclusions [15]. In clinical practice, surgeons urgently need a more targeted list of risk factors verified by rigorous methodology to facilitate precise risk stratification before surgery and adopt targeted preventive strategies during and after the operation [16, 17]. However, comprehensive studies that integrate injury characteristics, patients' baseline conditions, surgical details, and systematically control confounding effects are still insufficient.

This study therefore aimed to: (1) identify clinical and injury-related factors associated with specific postoperative complications after DRF surgery using PSM; (2) quantify the functional impact of complications on wrist motion, grip strength, and DASH scores; and (3) examine the directionality of the association between rehabilitation timing and complications, acknowledging the potential for reverse causality.

Based on the aforementioned clinical needs and the gap in existing evidence, this study aimed to conduct a retrospective observational analysis. While the association between prolonged operative time and complications has been reported, previous studies often lacked rigorous adjustment for case mix, combined heterogeneous complications, or did not quantify functional impact. This study therefore aimed to: (1) use propensity score matching to balance baseline confounders and estimate the association between operative time and a predefined set of specific complications; (2) quantify the effect of these complications on wrist function (range of motion, grip strength, DASH) at 6 months; and (3) examine the directionality of the rehabilitation–complication association, explicitly testing for reverse causality. By doing so, we provide a more cautious but robust estimate and identify key unmeasured confounders (e.g., soft tissue injury, surgeon experience) that should be prioritized in future prospective studies.

## Object and method of research

### Research subjects

**Fig. 1:**
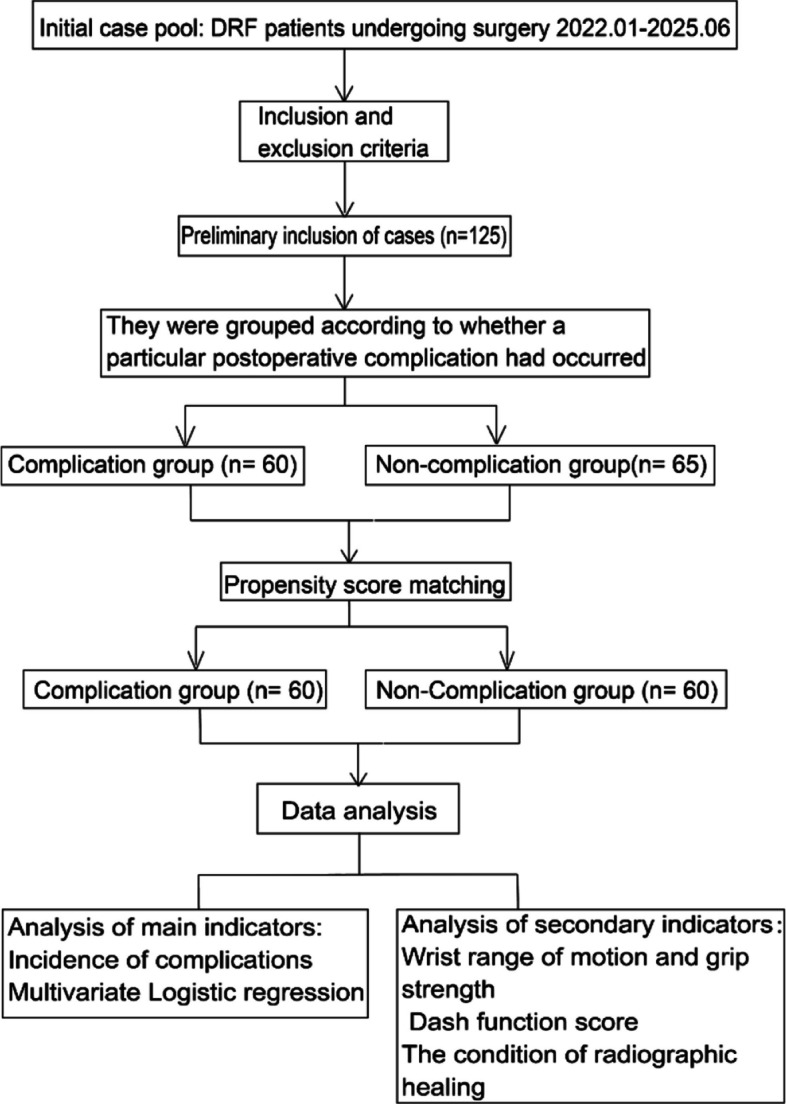
Flow chart of this study

This study adopted a retrospective observational design. The study subjects were adult patients with DRF who underwent surgical treatment at the orthopedic center of our hospital from January 1, 2022, to June 30, 2025. The study cases were identified and screened through the hospital's electronic medical record system and imaging archiving system. Cases with specific postoperative complications were defined as the "complication group", and cases without complications from the same period were matched to form the "no-complication group". To control confounding bias, this study employed the propensity score matching method, and at a 1:1 ratio, to ensure comparability of key baseline characteristics between the groups. A total of 138 consecutive patients underwent DRF surgery during the study period. Of these, 125 patients met eligibility criteria and completed a 6-month follow-up. In this source cohort, 60 patients (case group) were identified as having at least one predefined postoperative complication. The remaining eligible patients without any complications served as a potential control group (*N* = 65). From this control group, 60 controls were selected using 1:1 propensity score matching. Finally, the complication group (*n* = 60) and the no-complication group (*n* = 60) were formed.This was a retrospective matched case–control study conducted at a single center. Patients who developed specific postoperative complications after surgical treatment of distal radius fractures between January 2022 and June 2025 were identified as cases (complication group). From the same source population of patients who underwent surgery but did not develop any of the predefined complications, controls were selected using propensity score matching (PSM) at a 1:1 ratio. The study design thus does not aim to estimate the absolute incidence of complications in the overall surgical population but rather to compare risk factors between patients with and without complications under balanced baseline conditions, as shown in Fig. 1.

### Exclusion and inclusion criteria

#### Inclusion criteria

Li and Liu 2025 [18] (1) Age ≥ 18 years; (2) Diagnosed as fresh closed or open Gustilo type I or II distal radius fractures through imaging; (3) Treated with open reduction and internal fixation during the study period; (4) Having at least 6 months of complete follow-up records after the surgery.

#### Exclusion criteria

Zhang et al. 2025 [19] (1) Combined fractures in other parts of the same side upper limb; (2) Combined nerve and vascular injuries; (3) Pathological fractures; (4) Previous history of wrist surgery or severe deformity; (5) Accompanied by neurological or mental disorders that severely affect rehabilitation; (6) Patients for whom effective assessment cannot be conducted due to severe lack of data.

### Observation indicators

#### Key indicators

The primary indicators focus on the identification of complications and their associated risk factors. Complications are clearly defined as specific clinical events that occur within 6 months after surgery, including: 1) complex local pain syndrome (CRPS), diagnosed according to the Budapest clinical diagnostic criteria [20]; 2) tendon irritation or rupture with clinical symptoms, manifested as local pain, clicking, or active dorsiflexion dysfunction [21]; 3) carpal tunnel syndrome, defined as typical nerve compression symptoms that appear after surgery and are confirmed by objective examination. The typical symptoms include: numbness or pain on the radial side of the thumb, index finger, middle finger, and ring finger that occurs at night or in the early morning, often causing patients to wake up from sleep; repeated flexion and extension of the wrist joint can trigger or aggravate the above sensory abnormalities; patients may complain of clumsiness and weakness in the hand, especially when performing fine movements such as buttoning buttons; severely, there may be atrophy of the thenar muscle. Physical examination can reveal sensory loss in the median nerve innervation area (the above fingers), positive Tinel sign (numbness induced by tapping the wrist tunnel), and positive Phalen test (symptoms caused by sustained wrist flexion for 60 s) [22]. Diagnosis requires reliance on nerve conduction tests, showing that the sensory or motor conduction latency of the wrist segment of the median nerve is significantly prolonged compared to the healthy side, or there is a difference compared to the ipsilateral ulnar nerve [23]; 4) discomfort caused by complications related to internal fixation that requires secondary treatment; 5) nonunion (clear fracture line after surgery without signs of healing) or symptomatic malunion (loss of radial height > 2 mm, palmar inclination angle > 10 degrees, dorsal deviation or ulnar deviation angle < 15 degrees) [24]. The main analysis will calculate the incidence of overall and various complications and explore their independent risk factors.

Superficial or deep surgical site infections were not included as a specific complication in this analysis, because their incidence was extremely low (< 1%) in our source cohort and they were considered to have a distinct pathogenesis unrelated to the injury and surgical factors of primary interest.

#### Secondary indicators

1. Functional assessment 6 months after surgery

This study employed a combination of objective measurements and standardized patient self-assessment scales to conduct a comprehensive functional assessment at the critical time point of 6 months after surgery. The measurement of wrist joint range of motion was carried out by a fixed rehabilitation therapist using a standard long-arm digital angle measuring instrument. The patient was seated, with the shoulder joint abducted to 0, the elbow joint flexed to 90, and the forearm placed in a pronated position on the table. The maximum active palmar flexion, dorsal extension, radial deviation, and ulnar deviation angles of the wrist joint were measured in each direction [25]. Each direction was measured three times, and the average value was recorded. The unit was degrees. Grip strength measurement was conducted using a calibrated Jamar grip strength gauge (model FEI), with the handle set at the second position. Measurement standardization: The patient sat upright, with the shoulder retracted, the elbow flexed to 90, and the forearm in a neutral position. The patient was instructed to grip with maximum force, and each hand was tested alternately three times, with an interval of 30 s each time [26]. Record the maximum values of the affected side and the healthy side, and finally express it as "the percentage of the affected side's grip strength compared to the healthy side's grip strength" to eliminate the influence of individual absolute strength differences. The patient's self-assessment of function is evaluated using the Chinese version of the Upper Extremity Function Rating Scale (Disabilities of the Arm, Shoulder and Hand, DASH) [27]. This scale contains 30 core questions, covering symptoms of the upper limbs (pain, tingling, weakness) and the difficulty in performing various daily activities, work, and entertainment in the past week. Each question uses a 5-point Likert scale (1 point: no difficulty/no symptoms; 5 points: unable to complete/extremely severe). The final score is converted into a standardized score ranging from 0 to 100 through a formula, with a higher score indicating more severe functional impairment. The scale has good reliability and validity and has been widely used in upper limb function assessment.

(2) Factors related to injury

The AO (Arbeitsgemeinschaft für Osteosynthesefragen) classification of fractures was independently interpreted by two senior orthopedic physicians in a blinded manner based on preoperative or postoperative standard anteroposterior X-ray films and CT scans (if any). The final classification was based on the AO/OTA (Arbeitsgemeinschaft für Osteosynthesefragen / Orthopaedic Trauma Association) classification system: Type A (fractures outside the joint), Type B (partial intra-articular fractures), Type C (complete intra-articular fractures). Disagreements were resolved through negotiation or by a third expert. Whether it was an open fracture and its severity were retrospectively diagnosed according to the Gustilo-Anderson classification criteria, mainly based on the description of wound length, contamination degree, and soft tissue injury in the emergency medical records. This study only included Type I and Type II open fractures. The time from injury to surgery was defined as the interval from the recorded definite injury time to the start time of the surgical incision, calculated in hours, and precisely extracted from the anesthesia record sheet in the medical record system.

(3) Surgical-related factors: The surgical approach was clearly classified based on the surgical records as follows: palmar approach (dominantly the Henry approach), dorsal approach, combined approach, or others. The surgical time was defined as the total duration from skin incision to skin suture completion, measured in minutes, and the data were derived from the anesthesia record sheet. The use of internal fixation was recorded by the main type of internal fixation device (such as palmar locking plates, dorsal plates, external fixation frames, Kirschner wires, etc.), and whether it was simple internal fixation or combined with other fixation methods was also recorded.
(4) Postoperative complications: The diagnostic criteria for diabetes are defined as having a clear history of diabetes and undergoing continuous treatment (oral hypoglycemic drugs or insulin), or having a fasting blood glucose level ≥ 7.0 mmol/L or a glycated hemoglobin level ≥ 6.5% during hospitalization [28, 29]. The diagnostic basis for osteoporosis is: having a clear diagnosis of osteoporosis and receiving anti-osteoporosis treatment, or having measured bone density through dual-energy X-ray absorptiometry within the past year, with a T-value of ≤ -2.5 in any part (lumbar spine or hip) [30]. Relevant information is obtained by reviewing the patient's previous medical history records, medication list, and imaging reports.
(5) Postoperative management (braking period): The initial postoperative braking period is defined as the number of days from the end of the surgery to the start of the first standardized, unprotected wrist joint active or assisted active exercise. This information is determined by comprehensively reviewing postoperative orders, rehabilitation consultation records, and the first rehabilitation treatment records. Protective activities performed while wearing removable braces are not included in this period.
(6) Rehabilitation plan: The rehabilitation plan is recorded in three categories based on its start time, frequency, and content: 1) Early systematic rehabilitation: Intervention by a rehabilitation therapist within 2 weeks after the surgery, formulation and supervision of an individualized, progressive functional exercise plan; 2) Delayed or basic guidance: Regular rehabilitation treatment begins after 2 weeks after the surgery, or only oral and written guidance is given at discharge without continuous supervision; 3) No clear guidance: No systematic rehabilitation guidance or treatment records are found in the medical records. The extraction of rehabilitation information is mainly based on the consultation sheet of the rehabilitation department, treatment records, and discharge orders.

Furthermore, for conducting propensity score matching and multi-factor analysis, we defined detailed candidate variable indicators for data collection, covering multiple dimensions: demographic data (age, gender, dominant hand, injured side); injury-related factors (injury mechanism, fracture AO classification, whether it is an open fracture and its Gustilo classification, whether there is a ulnar styloid fracture and its displacement degree); patient's basic condition (smoking history, diabetes, diagnosis of osteoporosis or bone density T value); surgical-related details (time from injury to surgery, surgical approach, operation duration, type of internal fixation, whether concurrent carpal tunnel release was performed); and postoperative management (initial immobilization time, time to start standardized rehabilitation treatment). All imaging parameters and physical examination measurements were independently evaluated by two researchers, and differences were resolved through negotiation to ensure the objectivity and accuracy of the data.

### Sample size calculation

This was a retrospective study, and the sample size was determined by the number of eligible patients who developed specific postoperative complications during the study period. A total of 60 patients (case group) met the inclusion criteria and had complete follow‑up data. All 60 cases were included. From the pool of eligible patients without any complication (*n* = 65), a 1:1 matched control group (*n* = 60) was selected using propensity score matching. Consequently, the final analytic sample comprised 120 patients (60 cases, 60 controls).

No a-priori sample size calculation was performed, as the number of cases was fixed by the observed complication events. To assess whether this sample size provided adequate statistical power for the planned multivariate logistic regression (which included four independent variables), we conducted a post‑hoc power analysis. Assuming a two‑sided α = 0.05, a complication prevalence of 20% in the reference category, and the observed odds ratio for operative time (OR = 4.006), the achieved power was approximately 75%. However, for smaller effect sizes (e.g., OR = 2.0), the power dropped below 60%. Thus, the study was adequately powered to detect large effects but had limited power for moderate‑sized associations. This limitation is addressed in the Discussion.

### Statistical methods

Propensity score matching (PSM) was performed to minimise selection bias and balance baseline covariates between the complication and non-complication groups. The propensity score was estimated using a logistic regression model with complication status (yes vs. no) as the dependent variable. The following baseline covariates were included in the model: age (continuous), sex, AO fracture classification (A/B/C as ordinal), open fracture status (yes/no), and diabetes (yes/no). Matching was performed using 1:1 nearest-neighbour matching without replacement, with a caliper width set at 0.2 times the standard deviation of the logit of the propensity score. Patients in the complication group were matched to controls in the non-complication group from the eligible pool. After matching, balance was assessed using standardised mean differences (SMD) for all baseline covariates; an SMD < 0.1 was considered indicative of good balance. Additionally, we compared the distributions of covariates between groups using paired t-tests for continuous variables and McNemar’s tests for categorical variables to confirm balance (results shown in Table 1). Operative time was not included as a matching variable because it was the primary exposure of interest; including it would have resulted in over-matching and would have precluded assessment of its independent effect on complications.

**Table 1:** Comparison of baseline characteristics between the two groups after propensity score matching

Characteristic	Complication group (*n* = 60)	Non-complication group (*n* = 60)	Effect size (95% CI)	*P* value
Demographic data				
Age (years), mean ± SD	62.50 ± 11.83	60.13 ± 10.49	Cohen’s d = 0.21 (-0.15 to 0.57)	0.249
Sex, female, n (%)	46 (76.7)	44 (73.3)	Cramér’s V = 0.04	0.673
Dominant hand injury, n (%)	31 (51.7)	30 (50.0)	Cramér’s V = 0.02	0.855
Injury-related factors				
AO fracture classification, n (%)			Cramér’s V = 0.17	0.918
Type A	15 (25.0)	17 (28.3)		
Type B	20 (33.3)	19 (31.7)		
Type C	25 (41.7)	24 (40.0)		
Open fracture, n (%)	6 (10.0)	5 (8.3)	Cramér’s V = 0.10	0.752
Time from injury to surgery (days), mean ± SD	4.18 ± 2.49	3.93 ± 2.14	Cohen’s d = 0.11 (-0.25 to 0.47)	0.540
Comorbidities				
Diabetes mellitus, n (%)	12 (20.0)	10 (16.7)	Cramér’s V = 0.04	0.637
Osteoporosis, n (%)	18 (30.0)	15 (25.0)	Cramér’s V = 0.06	0.540
Surgical factors				
Volar approach, n (%)	55 (91.7)	56 (93.3)	Cramér’s V = 0.03	0.729
Operative time (minutes), mean ± SD	98.63 ± 25.37	85.31 ± 21.68	Cohen’s d = 0.57 (0.20 to 0.94)	**0.002**

Continuous variables were compared using t-test or Mann–Whitney U test; categorical variables using chi-square or Fisher’s exact test. Effect sizes: Cohen’s d (small ≥ 0.2, medium ≥ 0.5, large ≥ 0.8) and Cramér’s V (small ≥ 0.1, medium ≥ 0.3, large ≥ 0.5). Matching variables (age, sex, AO classification, open fracture status, diabetes) were well balanced after PSM (SMD < 0.1 for all). Operative time was intentionally excluded from the matching model as the primary exposure and, as expected, remained significantly different between groups by design (*p* = 0.002)

For the matched data, descriptive statistics and intergroup comparisons were performed. Continuous variables following a normal distribution (assessed by Shapiro–Wilk test) were expressed as mean ± standard deviation and analysed using the independent samples t-test; non-normally distributed data were described by median and interquartile range and analysed using the Mann–Whitney U test. Categorical variables were expressed as frequency and percentage and analysed using the chi-square test or Fisher’s exact test as appropriate.

For the primary outcomes, variables were selected a priori based on clinical relevance and univariate analysis (*p* < 0.10). The following four variables were forced into the multivariate logistic regression model using the enter method (no stepwise selection was applied): age (dichotomised as ≥ 60 vs. < 60 years), operative time (> 90 vs. ≤ 90 min), AO fracture classification (type C vs. types A/B), and diabetes (yes vs. no). Open fracture status was not included in the multivariable model due to the small number of events (*n* = 11 in the matched source cohort). The model’s goodness-of-fit was assessed using the Hosmer–Lemeshow test, and multicollinearity was checked using variance inflation factors (VIF). Adjusted odds ratios (OR) with 95% confidence intervals (CI) were calculated.

For the secondary outcomes involving repeated measurements (DASH scores assessed at 1, 3, and 6 months postoperatively), a two-way repeated-measures analysis of variance (ANOVA) was conducted, with group (complication vs. non-complication) as the between-subjects factor and time (T1, T2, T3) as the within-subjects factor. The sphericity assumption was checked using Mauchly’s test; if violated, the Greenhouse–Geisser correction was applied. The primary outcomes of interest were the main effects of group and time, and the group × time interaction. Post-hoc pairwise comparisons with Bonferroni correction were performed for significant interaction effects. Partial eta squared (η^2^p) was reported as the effect size measure for all ANOVA effects.

All statistical analyses set the two-sided significance level α = 0.05.

### Ethical statement

This study was approved by the Institutional Review Board of Zhangjiagang First People’s Hospital (Approval No. Zjgyyll-Kysb-2025–11-004). Because this was a retrospective observational study involving only anonymized extraction and analysis of pre‑existing medical records, with no direct patient contact and no modification of clinical practice, the IRB granted a waiver of informed consent in accordance with the principles of the Declaration of Helsinki and relevant national regulations. All data were de‑identified prior to analysis, and patient confidentiality was strictly protected throughout the study.

## Results

### Baseline characteristics

After propensity score matching (Table 1), most baseline characteristics—including age, gender, fracture AO classification, proportion of open fractures, prevalence of diabetes, and osteoporosis—showed no statistically significant differences between the two groups (*p* > 0.05, SMD < 0.1), indicating successful matching and good group comparability. The standardized mean differences (SMD) for all matched covariates ranged from 0.025 to 0.112, all below the prespecified threshold of 0.1. The only exception was operative time, which was intentionally excluded from the propensity score model to avoid over‑matching and to allow evaluation of its independent association with complications in subsequent regression analysis. As expected, operative time remained significantly longer in the complication group than in the group without complications after matching (*p* = 0.002).

### Patient-specific proportion of cases with specific complication types

Table 2 presents the distribution of specific complication types among the 60 patients in the matched case group. All patients experienced at least one predefined complication. The most frequent subtypes were tendon irritation/rupture (19/60, 31.7%) and internal fixation-related discomfort (14/60, 23.3%), followed by carpal tunnel syndrome (12/60, 20.0%) and CRPS (8/60, 13.3%). Non-union/malunion occurred in 7 patients (11.7%). Because some patients had more than one complication, the sum of percentages exceeds 100%.

**Table 2:** Distribution of specific complication types within the case group

Complication type	Number of patients (*n* = 60)	Proportion (%)
Tendon irritation/rupture	19	31.7
Internal fixation-related discomfort	14	23.3
Carpal tunnel syndrome	12	20.0
Complex regional pain syndrome (CRPS)	8	13.3
Non-union/malunion	7	11.7

CRPS is Complex local pain syndrome.Some patients had more than one complication type, so the sum of proportions exceeds 100%

### Multivariate Logistic regression analysis

According to the results of the multivariate Logistic regression analysis in Table 3, among all the candidate variables included in the model, only "surgery duration > 90 min" was identified as an independent risk factor for the occurrence of specific postoperative complications, with a statistical significance of *p* = 0.001.

**Table 3:** Multivariate Logistic regression analysis of the risk of postoperative complications

Parameters	β	Standard error	Wald χ^2^	*df*	*OR*	*OR 95% CI*	*p* -value
Age ≥ 60 years	0.352	0.407	0.749	1	1.422	0.641, 3.155	0.387
Operative Time > 90 min	1.388	0.407	11.650	1	4.006	1.806, 8.890	0.001
Fracture Type AO-C	0.346	0.408	0.719	1	1.413	0.635, 3.146	0.397
Diabetes (Yes)	0.549	0.513	1.146	1	1.731	0.634, 4.729	0.284
Constant	-1.115	0.446	6.236	1	0.328		0.013

*OR* Odds ratio, *CI* Confidence interval, *AO* Arbeitsgemeinschaft für Osteosynthesefragen

Specifically, after adjusting for other factors such as age, fracture type, and diabetes, patients with an operative time exceeding 90 min had a significantly higher odds of complications (OR = 4.006, 95% CI: 1.806–8.890, *P* = 0.001). However, operative time may serve as a proxy for unmeasured aspects of case complexity (e.g., fracture comminution severity, soft tissue injury, surgical difficulty) that were not fully captured by the matching variables. Therefore, this association should be interpreted as a marker of increased risk rather than a direct causal factor. The Hosmer–Lemeshow test indicated good model fit (χ^2^ = 6.32, df = 8, *p* = 0.61), and variance inflation factors for all included variables were below 2, suggesting no significant multicollinearity.

### Secondary outcomes

According to the results in Table 4, the objective functional assessment at 6 months after surgery showed that the patient group with specific complications (complication group) had significantly worse recovery in wrist joint range of motion and grip strength compared to the group without complications (no complications). All the differences in the comparison items were highly statistically significant (*p* < 0.001).

**Table 4:** Comparison of wrist range of motion and grip strength at 6 months postoperatively

Activity	Complication group (*n* = 60)	Non-complication group (*n* = 60)	Test	95% CI		F	t	*p* -value
				**Lower**	**Upper**			
Palm flexion	52.31 ± 11.47	65.84 ± 9.21	t test	-17.281	-9.760	4.733	-7.119	< .0.001
Back stretch	48.62 ± 12.06	62.08 ± 8.74	t test	-17.269	-9.651	7.864	-6.997	< .0.001
Radial deviation	18.17 ± 8.28	22.91 ± 4.13	t test	-7.104	-2.376	39.011	-3.971	< .0.001
Ulnar deviation	25.43 ± 7.04	31.61 ± 5.48	t test	-8.461	-3.899	1.241	-5.365	< .0.001
Grip strength (%)	78.49 ± 15.19	92.33 ± 12.84	t test	-18.923	-8.757	0.193	-5.392	< .0.001

In terms of wrist joint range of motion, the complication group had significantly restricted active range of motion in all directions. Specifically, the average flexion angle decreased by approximately 13.5 degrees (52.3 vs 65.8), and the average extension angle decreased by approximately 13.5 degrees (48.6 vs 62.1). The ulnar deviation and radial deviation activities also decreased by an average of 4.7 degrees (18.2 vs 22.9) and 6.2 degrees (25.4 vs 31.6), respectively.

In terms of grip strength recovery, the recovery situation of the complication group was also not ideal. The grip strength on the affected side only recovered to 78.5% of the healthy side, while the no-complication group recovered to 92.3%, with a difference of 13.8 percentage point.

### DASH score

The DASH scores at 1, 3, and 6 months postoperatively are presented in Table 5. A two-way repeated-measures ANOVA revealed a significant main effect of time (F = 303.08, *p* < 0.001, η^2^p = 0.72), indicating that DASH scores improved significantly over the follow-up period in both groups. There was also a significant main effect of group (F = 61.42, *p* < 0.001, η^2^p = 0.34), with the non-complication group having consistently lower (better) DASH scores than the complication group. Importantly, a significant group × time interaction was observed (F = 5.91, *p* = 0.018, η^2^p = 0.05), indicating that the rate of improvement differed between the two groups. Post-hoc comparisons with Bonferroni correction showed that the complication group had significantly higher DASH scores at 3 months (mean difference 12.57, 95% CI: 6.21–18.93, *p* < 0.001) and at 6 months (mean difference 12.75, 95% CI: 7.31–18.19, *p* < 0.001), but not at 1 month (mean difference 2.29, 95% CI: -2.58 to 7.16,* p* = 0.63).

**Table 5:** Comparison of DASH scores at different postoperative time points

Activity	T1 (1 month)	T2 (3 months)	T3 (6 months)	Test	Difference 95% CI	F	η^2^p	*p-*value
Complication group (*n* = 60)	65.42 ± 12.81	42.74 ± 15.31	28.51 ± 13.62	t test	-17.281 to -9.760	61.419	0.342	< 0.001
Non-complication group (*n* = 60)	63.13 ± 11.49	30.17 ± 12.41	15.76 ± 10.24	t test				

F_group = 61.419, *p*_group < 0.001; F_time = 303.084, p_time < 0.001; F_group × time = 5.914, p_group × time = 0.003

T1 = 1 month postoperatively, T2 = 3 months postoperatively, T3 = 6 months postoperatively.Data are presented as mean ± SD. DASH = Disabilities of the Arm, Shoulder and Hand; higher scores indicate worse function. Repeated-measures ANOVA revealed a significant group × time interaction (F = 5.914, *p* = 0.018). All 120 matched patients completed the 6-month follow-up with no missing DASH data

### Association between AO classification of different fractures and major complications

The incidence rates of the three major complication subtypes increased with higher AO fracture grades (Table 6). Specifically, tendon complications occurred in 6.3% of Type A, 15.4% of Type B, and 20.4% of Type C fractures (*p* for trend = 0.042). Carpal tunnel syndrome rates were 3.1%, 10.3%, and 14.3%, respectively (*p* for trend = 0.046). Osseous complications (non-union/malunion) occurred in 0%, 5.1%, and 10.2%, respectively (*p* for trend = 0.025).

**Table 6:** Association between AO fracture classification and major complication subtypes

Types of complications	Type AO-A (*n* = 32)	Type AO-B (*n* = 39)		Type AO-C (*n* = 49)	Test	Effect size	F		*df*	*p* -value
Complications of tendon	2(6.25)		6 (15.38)	10 (20.41)	Chi-squared t	0.174		4.126	2	0.042
Carpal tunnel syndrome	1(3.13)		4 (10.26)	7 (14.29)	Chi-squared t	0.127		3.982	2	0.046
Osseous complications	0 (0.00)		2 (5.13)	5 (10.20)	Chi-squared t	0.150		5.017	2	0.025

CRPS and internal fixation-related discomfort were not included in this stratified analysis because their occurrence was not sufficiently correlated with AO grade in the preliminary univariate analysis (*p* > 0.05 for trend). The three subtypes shown had a clear graded association

The results showed that the incidence rates of the three main complications all presented a significant increasing trend as the fracture classification level increased. Specifically, the incidence rates of tendon complications (including irritation and rupture) were 6.3%, 15.4%, and 20.4% in Types A, B, and C, respectively, and the trend test p-value was 0.042. The incidence rate of carpal tunnel syndrome increased from 3.1% in Type A to 14.3% in Type C, and the trend was also significant (*p* = 0.046). In terms of bony complications (nonunion/malunion reflecting bone healing problems), this trend was more obvious. No cases occurred in Type A patients, 5.1% in Type B, and 10.2% in Type C, with a trend p-value of 0.025.

### Distribution of types of postoperative rehabilitation programs

According to the results in Table 7, this study compared the rehabilitation programs received by the two groups of patients after surgery, and found that there were significant differences in the timing of rehabilitation intervention and the system, and this difference was clearly associated with the occurrence of complications.

**Table 7:** Distribution of types of postoperative rehabilitation programs

Rehabilitation programme	Complication group	Non-complication group	Test	Effect size	F	*df*	*p* -value
Early systemic rehabilitation	18 (30.00)	35 (58.33)	Chi-squared t	0.293	10.335	2	0.006
Delay or basic instruction	30 (50.00)	20 (33.33)					
No clear direction	12 (20.00)	5 (8.33)					

The proportion of patients who received “early systematic rehabilitation” was significantly higher in the non-complication group compared with the complication group (58.3% vs. 30.0%, *p* = 0.003). While this association is statistically significant, the retrospective design precludes any determination of causality. It remains equally plausible that early complications (e.g., pain, stiffness, tendon irritation) led to delayed rehabilitation, rather than early rehabilitation preventing complications. Therefore, these findings should be interpreted as exploratory and hypothesis-generating only, not as evidence of a protective effect of early rehabilitation.

## Discussion

Throughout the following discussion, we distinguish between direct observations from our data (e.g., adjusted odds ratios, group comparisons) and speculative explanations of underlying mechanisms. The latter are drawn from the literature and are presented as hypotheses to guide future research, not as conclusions proven by this study.

The core findings of this study highlight the complexity and interplay of factors associated with specific complications after distal radius fracture surgery. After controlling for baseline confounders through propensity score matching, we identified prolonged surgery time as an independent risk factor, while the initial severity of the fracture (AO-C type) and the lack of early systematic rehabilitation jointly constituted the risk background and potential intervention gap for complications. These results are not isolated; they are interwoven, pointing to a more complex clinical picture — the occurrence of complications is often the result of the combined effects of patient factors, injury characteristics, surgical quality, and postoperative management. It is important to note that the following discussion distinguishes between directly observed associations (e.g., odds ratios, group comparisons) and speculative mechanistic explanations drawn from the literature, which were not directly tested in this study. Examining our findings within the existing literature framework not only verifies their reliability but also generates hypotheses for future mechanistic and interventional research.

In brief, our main findings are: (i) operative time > 90 min showed the strongest association with complications (OR 4.01), but this association is likely confounded by unmeasured case complexity; (ii) AO-C fractures and lack of early systematic rehabilitation were also associated with higher complication rates, yet these associations may be non-causal due to residual confounding or reverse causality; (iii) complications led to significantly worse functional outcomes at 6 months. Thus, the primary contribution of this observational study is not to claim causal risk factors, but to provide a cautiously estimated association and to highlight the crucial unmeasured confounders that must be addressed in future prospective studies.

Comparison with previous literature and unique contributions of this study The association between longer operative time and higher complication risk after DRF surgery has been reported in several retrospective series (e.g., Muder et al. [31]). However, most previous studies either did not use propensity score matching to balance baseline covariates, grouped dissimilar complications together, or did not systematically assess functional outcomes. Our study adds to the existing knowledge in several ways. First, by applying PSM, we reduced confounding from age, sex, fracture classification, and comorbidities, providing a less biased estimate of the operative time–complication association. Second, we focused on five specific complications with explicit definitions and reported their distribution separately, avoiding the “lumping” that often obscures risk heterogeneity. Third, we quantified the functional deficit at 6 months after complications, showing an approximately 13–14% reduction in grip strength and significantly worse DASH scores. Fourth, we explicitly addressed reverse causality in the rehabilitation analysis, demonstrating that the apparent “protective effect” of early rehabilitation may be entirely due to residual confounding or bidirectional relationship – a cautionary finding for clinicians who might otherwise overinterpret observational data. Finally, we transparently listed unmeasured confounders (soft tissue injury, surgeon experience, implant positioning) that likely drive the operative time effect, guiding future prospective studies to collect these variables. Thus, while our findings are not mechanistically novel, they provide a methodologically rigorous confirmation and extension of existing knowledge, with direct implications for the design of future studies and for cautious clinical interpretation.

The duration of surgery exceeding 90 min emerged as the strongest independent predictor. This finding aligns with the conclusions of several studies, but its prominent position in the multivariate model of this study merits in-depth analysis. Perregaard et al., in a study involving 300 patients with volar plate fixation, noted that for every 30‑minute increase in surgical duration, the overall postoperative complication risk increased by approximately 1.5 times, an effect they mainly attributed to the longer operative time required for complex fractures [32]. However, our analysis, after controlling for AO fracture classification, still showed a significant independent association between operative time and complication risk. This suggests that operative time itself may confer additional risk beyond fracture complexity.While our study did not directly investigate the underlying biological mechanisms, several hypotheses can be proposed based on the literature. One speculative explanation is that prolonged surgery may involve more extensive soft tissue dissection, longer traction, and longer tourniquet use. If confirmed in future studies, these factors could potentially exacerbate local ischemia‑reperfusion injury, tissue edema, and inflammatory responses, which might in turn predispose to tendon adhesion, nerve irritation, and pain syndromes [33, 34]. This is consistent with the observations of Chen's team, who found through intraoperative tissue oxygen monitoring that the duration of surgery was positively correlated with the degree of decrease in wrist soft tissue oxygen saturation, and the latter was related to early postoperative pain scores. [35]. These mechanistic pathways remain hypothetical and require direct experimental or intraoperative validation.

A critical limitation of our analysis is that operative time likely reflects underlying case complexity rather than exerting a direct biological effect. Despite adjusting for AO classification, open fracture status, and diabetes through PSM and multivariable regression, substantial residual confounding may remain. Factors not measured in our retrospective database—such as the degree of soft tissue injury, intraoperative bleeding, number of reduction attempts, surgeon experience and skill, and implant selection—could simultaneously prolong operative time and increase complication risk. Consequently, the observed association between operative time and complications may be confounded by these unmeasured variables. We therefore caution against overinterpreting operative time as an independent causal risk factor. Instead, it should be viewed as a composite indicator of surgical complexity and potentially modifiable process measure. Future studies should collect granular intraoperative data (e.g., video analysis, difficulty scoring systems) or employ instrumental variable methods to better isolate the effect of operative time from case mix.

Regarding AO fracture classification, our study found a significant association between Type C fractures and a higher frequency of complications, particularly tendon issues and non-union/malunion. This finding is consistent with extensive literature and supports current clinical consensus. For example, Chao et al. reported that C-type fractures correlated with significantly lower functional DASH scores (mean 16.0) compared with A-type (11.3) and B-type (11.9) fractures (ANOVA, *p* < 0.05) [36]. Our trend analysis further quantified this risk gradient, showing that the incidence of the three major complication subtypes increased almost linearly from Type A to Type C (Table 6).

One plausible explanation, though not directly tested in our study, is that Type C fractures involve joint fragmentation, metaphyseal comminution, and extensive ligamentous injury. Such features might challenge reduction stability and fixation, and could be associated with more severe soft tissue trauma and periarticular vascular damage [37, 38]. However, these mechanistic interpretations remain speculative based on our observational data. It is worth noting that in our multivariable model, AO-C type and age ≥ 60 years did not reach statistical significance, whereas operative time did. This finding does not imply that fracture severity or age are unimportant. One hypothesis is that Type C fractures and older age may operate partly through prolonging surgical time—i.e., collinearity may exist. If so, surgical time, as a more immediate intraoperative variable, could absorb some of the explanatory power. This speculation aligns with the observation by Nguyen et al. that surgical complexity indicators may have higher predictive efficacy than static fracture classifications when both are included [39]. Nevertheless, formal mediation analysis or larger sample sizes would be required to test this hypothesis.

Given the unresolved directionality, the association between rehabilitation timing and complications should not be interpreted as causal. This analysis serves primarily to generate hypotheses for future prospective studies. The observed association between postoperative rehabilitation protocols and complication frequency represents another key finding of this study. Our data show that patients who received “early systematic rehabilitation” had a significantly lower proportion of complications compared with those who did not (58.3% vs. 30.0% in the non‑complication and complication groups, respectively). This finding is consistent with results from studies on enhanced recovery after surgery (ERAS) protocols in orthopedics [40, 41]. The potential benefits of early, active, and supervised rehabilitation are well recognized: it may reduce postoperative edema, prevent joint capsule and ligament contracture, promote tendon gliding, and improve proprioception and motor control through neuromuscular re‑education [42, 43]. Several randomized controlled trials support the efficacy of early mobilization. For example, Quadlbauer et al. demonstrated that for patients with stable internal fixation, initiating protected active movement immediately after surgery resulted in better wrist range of motion and patient satisfaction at 6 months compared with traditional immobilization, without increasing the risk of fixation failure [44]. However, the retrospective design of our study precludes any causal interpretation of the association between rehabilitation timing and complications. It remains possible that reverse causality explains the observed relationship: patients who developed early complications (e.g., severe pain, tendon irritation, or marked stiffness) might have been less likely to receive or adhere to early systematic rehabilitation, rather than the absence of rehabilitation causing the complications. Unmeasured confounders—such as patient motivation, socioeconomic status, health literacy, or surgeon referral patterns—could also have influenced both rehabilitation assignment and complication risk. Therefore, while our findings generate the hypothesis that early systematic rehabilitation may reduce complications, they do not establish causality. The term “systematic” in rehabilitation refers to individualized, progressive plans with regular assessment and adjustment, which differs from simple discharge instructions. Prospective studies with predefined rehabilitation protocols and time‑to‑event analyses are needed to determine whether early systematic rehabilitation directly lowers complication rates or merely serves as a marker for better overall care.

By integrating the aforementioned core factors—surgical technique efficiency, severity of fracture, and quality of rehabilitation—we were able to construct a more comprehensive complication risk model. Elderly patients, due to possible osteoporosis (which increases the risk of type C fractures), decreased tissue healing ability, and reduced tolerance to surgical trauma, form the baseline of risks. When such patients suffer from high-energy injuries resulting in type C fractures, the surgical challenge doubles, and it is extremely likely to exceed the critical 90-min time window. If, after the surgery, due to various reasons (such as patient compliance, accessibility of medical resources, or lack of cognition), early systematic rehabilitation is not achieved, then the complication risk chain is fully formed. This study presents each key link on this chain through multivariate analysis and stratified comparison. This is consistent with the comprehensive analytical approach of Sellés et al., who called for the establishment of a multi-factor predictive model rather than relying on a single indicator.

Furthermore, our analysis of specific complication subtypes provides additional observations that align with previously reported mechanisms, although these mechanisms were not directly tested in our study. The high incidence of tendon complications (particularly extensor tendonitis) has been previously attributed to plate placement too proximal or screws penetrating the dorsal cortex when using the palmar approach, as demonstrated in anatomical and clinical studies by Soo et al. and Azad [45, 46]. Consistent with these reports, our finding of a high proportion of tendon complications (31.7% in the case group) indirectly supports the clinical relevance of such technical factors, though we did not collect detailed intraoperative measurements of screw position or plate location. Therefore, our results highlight the potential importance of multi‑angle intraoperative fluoroscopy to confirm screw length and the use of smooth‑edge plates to reduce tendon irritation—recommendations that derive from the literature rather than direct evidence from this study.

Regarding carpal tunnel syndrome, its occurrence has been linked to reduced carpal tunnel volume due to initial fracture displacement, intraoperative traction, and persistent postoperative swelling. The study by Demino et al. identified preoperative median nerve symptoms and high-energy injury mechanisms as risk factors [47]. Our study did not assess preoperative nerve status or carpal tunnel dimensions. Nevertheless, the strong association between AO-C fractures and carpal tunnel syndrome (14.3% in type C vs. 3.1% in type A, P for trend = 0.046) is consistent with the hypothesis that greater injury severity contributes to this complication. This interpretation remains speculative and requires confirmation in studies specifically designed to measure carpal tunnel pressures or nerve conduction parameters.

This study adopted propensity score matching in its methodology, which is an important innovation distinct from many previous retrospective studies. Most similar studies mostly employ direct grouping comparison or conventional regression to control confounding factors. The application of PSM, based on the principle of randomized controlled trials, constructed a more comparable group without complications in observational data, making the result estimation closer to the true causal relationship. For example, when comparing rehabilitation effects, it balanced factors such as age and fracture type that may affect the choice of rehabilitation plans, making the effect of the "rehabilitation plan" itself more prominent.

## Limitations

This study has several limitations. First, as a single-center retrospective observational study, its generalizability is limited because differences in surgical techniques, rehabilitation protocols, and patient populations across centers may affect risk factor weights. Second, although propensity score matching reduced confounding bias, inherent retrospective limitations remain. Important potential confounders—including surgeons’ technical preferences, socioeconomic status, smoking, implant positioning details (e.g., screw or plate prominence), and intraoperative factors (e.g., tourniquet time, soft tissue handling)—were unavailable in our database. Residual confounding from unmeasured variables may exist, requiring cautious interpretation of observed associations. Third, the analysis of rehabilitation protocols is subject to fundamental reverse causality that cannot be resolved retrospectively: we cannot determine whether delayed rehabilitation is a consequence or a cause of complications. Thus, the observed association provides no evidence for a protective effect of early rehabilitation; any such interpretation is speculative and requires prospective validation with predefined protocols and time-to-event analyses. Fourth, the sample size was determined by observed complication cases (*n* = 60) rather than a prospective power calculation. Although this meets the minimal requirement of 5–10 events per variable (4 variables, 60 events = 15/variable), the study was underpowered to detect moderate effect sizes (e.g., OR≈1.5–2.0). Post-hoc power analysis showed that for an OR of 2.0 with a risk factor prevalence of 20%, power was only 52%. Therefore, lack of significance for some clinically relevant variables (e.g., diabetes, AO-C type) may reflect type II error rather than true absence of association. Larger prospective studies are needed. Fifth, combining multiple complications in analysis may mask specific pathological mechanisms and risk spectra of different complications (e.g., tendinitis vs. CRPS). Subgroup and rare complication analyses remain underpowered. Sixth, operative time—our primary exposure—is likely a proxy for unmeasured case complexity. Although we adjusted for AO classification and open fracture status, these may not fully capture soft tissue injury, fracture instability, or technical difficulty. Thus, residual confounding remains a major concern, and the observed odds ratio for operative time should be interpreted cautiously. Finally, we acknowledge that our main finding (operative time associated with complications) replicates well-established knowledge. The value of this study lies not in discovering a new risk factor, but in providing a more rigorous estimate using PSM, quantifying functional consequences, and exposing critical residual confounders that must be measured in future research. Future prospective, multi-center studies with comprehensive biological, psychological, and social variables are needed to construct more accurate risk prediction models.

## Conclusion

In conclusion, this study confirms the strong association between prolonged operative time (> 90 min) and postoperative complications after DRF surgery, using propensity score matching to reduce confounding. However, this association may largely reflect unmeasured case complexity (e.g., soft tissue injury, surgeon experience) rather than a direct causal effect. The associations observed for fracture severity (AO-C) and early rehabilitation were either confounded or potentially bidirectional. The novel contributions of this study are the quantification of functional loss due to complications (13–14% grip strength reduction, worse DASH scores), the explicit demonstration of potential reverse causality in rehabilitation analyses, and the transparent listing of key unmeasured confounders to guide future prospective studies. Clinically, surgical teams should be aware that longer operative time serves as a marker of complex cases, which may require intensified perioperative monitoring rather than simply aiming to shorten surgery at the expense of quality.

## Data Availability

The data supporting the findings of this study can be obtained from the corresponding author, upon request.

## Abbreviations

ANOVA: Analysis of variance
AO: Arbeitsgemeinschaft für Osteosynthesefragen (German for "Association for the Study of Internal Fixation")
CI: Confidence interval
CRPS: Complex regional pain syndrome
DASH: Disabilities of the Arm, Shoulder and Hand
DRF: Distal radius fracture
ERAS: Enhanced recovery after surgery
IRB: Institutional Review Board
n: Number of patients
OR: Odds ratio
PSM: Propensity score matching
SD: Standard deviation
SMD: Standardised mean difference
T1, T2, T3: Time points: 1, 3, and 6 months postoperatively
VIF: Variance inflation factor
η^2^p: Partial eta squared
